# Reply 1: An assessment of the preconceptional mitochondrial hypothesis

**DOI:** 10.1038/sj.bjc.6600980

**Published:** 2003-05-27

**Authors:** M Sanderson, X O Shu, W Zheng

**Affiliations:** 1 University of Texas, Houston School of Public Health at Brownsville, Brownsville, TX 78520, USA; 2 Center for Health Services Research and Vanderbilt-Ingram Cancer Center, Vanderbilt University, Nashville, TN 37232-8300, USA

**Sir,**

We found Dr van Noord's preconceptional mitochondrial hypothesis interesting particularly in line with a recent report linking polymorphisms of two DNA base excision repair genes (XRCC1 and hOGG1) to breast cancer risk in daughters born to older mothers (Hodgson *et al*, 2003). Manganese superoxide dismutase (MnSOD) may impair the mitochondria's ability to reduce oxidative stress (Oberley and Oberley, 1997). MnSOD has been linked to breast cancer (Ambrosone *et al*, 1999), and may be another pathway through which older maternal age may function. Further support for this hypothesis comes from a recent study that found mitochondrial DNA damage in breast cancer tissue (Richard *et al*, 2000).

To test this hypothesis, we analysed the association of parental age with breast cancer risk using data from the Shanghai Breast Cancer Study (SBCS), and the results are in shown Table 1

**Table 1:** Odds ratios of breast cancer associated with maternal age and paternal age

	**Cases (*n*=288)**	**Controls (*n*=350)**	**OR^a^**	**(95% CI)**	**OR^b^**	**(95% CI)**
*Maternal age (years)*						
<25	73	98	1.0	(referent)	1.0	(referent)
25–29	123	127	1.4	(0.8–2.3)	1.6	(0.9–2.7)
30–34	63	77	1.1	(0.6–2.0)	1.5	(0.8–2.9)
⩾35	29	47	1.1	(0.5–2.2)	1.6	(0.7–3.9)
*P* trend			*P*=0.84		*P*=0.34	
						
*Paternal age (years)*						
<25	34	41	1.0	(referent)	1.0	(referent)
25–29	96	94	1.4	(0.7–2.8)	1.3	(0.6–2.7)
30–34	88	103	1.3	(0.7–2.7)	1.2	(0.6–2.6)
⩾35	70	111	0.9	(0.4–1.9)	0.7	(0.3–1.8)
*P* trend			*P*=0.13		*P*=0.08	

a Adjusted for age, income, family history of breast cancer in first-degree relative, history of fibroadenoma, age at menarche, parity, and age at first live birth, and pregnancy order.

b Adjusted for age, income, family history of breast cancer in first-degree relative, history of fibroadenoma, age at menarche, parity, age at first live birth, pregnancy order, and maternal age or paternal age.

. After adjustment for established breast cancer risk factors and pregnancy order, we did not find an association between older maternal or paternal age and premenopausal breast cancer in our low-risk population. Additional adjustment for paternal age resulted in a nonsignificantly elevated risk of breast cancer associated with older maternal age. All perinatal information was based on maternal report.

Although we collected information on whether the mother had a threatened miscarriage with the index pregnancy, too few women reported this adverse event (six case mothers, 13 control mothers) to provide a stable risk estimate. Other studies may have sufficient numbers of mothers to investigate this aspect of Dr van Noord's hypothesis.

Dr van Noord argued that insulin-like growth factor- I (IGF-1) might be unlikely to explain the inconsistent findings on birth weight and breast cancer risk in the literature, since the link between IGF-I and breast cancer risk has been found primarily in premenopausal women, while the high birth weight-breast cancer association has been seen among pre- and postmenopausal women. A previous report from the SBCS showed that elevated levels of IGF-I were associated with an increased risk of breast cancer among all women, but the association was more pronounced among women diagnosed premenopausally and among women with a high body mass index or waist-to-hip ratio (Yu *et al*, 2001). We found in a large US study that high birth weight was associated with an elevated risk among premenopausal women (OR=1.7, 95% CI 1.1–2.5), but a nonsignificantly reduced risk among postmenopausal women (OR=0.6, 95% CI 0.3–1.1) (Sanderson *et al*, 1996). Therefore, IGF-I as a potential explanation for the birth weight-breast cancer relationship cannot be ruled out.
